# Breast cancer cells exploit mitophagy to exert therapy resistance

**DOI:** 10.18632/oncotarget.24533

**Published:** 2018-02-19

**Authors:** Liana Hardy, Michele Frison, Michelangelo Campanella

**Affiliations:** Department of Comparative Biomedical Sciences, Royal Veterinary College, University of London, London, United Kingdom; University College London Consortium for Mitochondrial Research, University College London, London, United Kingdom; Department of Biology, University of Rome Tor Vergata, Rome, Italy; Regina Elena, National Cancer Institute, Rome, Italy

**Keywords:** mitochondria, mitophagy, chemotherapy, breast cancer

The understanding of mitochondria and their quality control in cancer genesis and progression is increasing exponentially. While the Warburg hypothesis depicts mitochondria as silent within a glycolytic tumorigenic environment, it is now known that in malignant cells mitochondria are constitutively active, priming malignant reprogramming and promoting survival. Thus, in cancer cells mitochondria supply energy, provide building blocks for new cells, control redox homeostasis and define oncogenic signals commanding programmed cell death [1]. Two other aspects of mitochondrial physiology have been relatively unexplored in the context of cancer pathogenesis: mitochondrial biogenesis and autophagic degradation for quality control. These two biological processes work together to regulate mitochondrial mass, morphology and function to accumulate or eliminate mitochondria for the purpose of cell progression. Furthermore, cross-analysis of the mitochondrial genomes in various cancers has revealed that there is a negative selection for pathogenic mtDNA mutations, suggesting an active engagement of mechanisms for mitochondrial removal within clinical cancers [1, 2]. The work by Biel and Rao highlights mitophagy as an efficient mechanism of chemotherapy resistance, demanding further attention on this process to inform innovative approaches of therapeutic value [3]. Targeting mitochondria as an anti-cancer strategy has historically provided obstacles to successful therapy. This is due to the development of resistance phenomena such as those ascribed to the exploitation of the mitochondrial retrograde response, questioning whether mitophagy should have the opposite effect.

Intrinsic aspects of mitochondrial function in cancer cells allow for the selective targeting of tumorigenic cells, including differences in mitochondrial membrane potential (Δψ_m_) which provides a putative target for anti-cancer therapy. Mitochondrial redox molecules conjugated to triphenylphosphonium (TPP) exploit such differences and accumulate within the mitochondria as a selective therapeutic approach.

Biel and Rao, describe how these TPP-conjugated antioxidants trigger a mitophagy-mediated resistance mechanism [3]. The role of mitophagy in tumorigenesis and cancer cell survival is likely to be context-dependent, controlled by the metabolic demands and stage of the tumour. Prior to neoplastic transformation, mitophagy is generally considered anti-tumorigenic, used by the healthy cell to selectively degrade dysfunctional mitochondria and prevent the accumulation of tumour-promoting reactive oxygen species (ROS) [4]. However, during conditions where mitochondria are chemically targeted to induce selective cellular demise mitophagy may act as a pro-survival mechanism, thereby removing the targets necessary for this strategic intervention [4].

The pharmacological control of mitophagy could in turn be tested as a way to improve the therapeutic efficacy of chemotherapy. Hitherto, few selective non-toxic inducers of mitophagy have been developed, i.e. Urolithin A [5] and the p62-mediated mitophagy inducer (PMI) [6] but pharmacological protocols to prevent mitophagy are yet to be developed. This could clearly be beneficial to aid therapy undermined by hyperactive mitophagy. Interestingly, anti-mitophagy molecules such as the mitochondrial translocator protein (TSPO) are overexpressed in aggressive forms of cancer implying that molecular regulation of mitophagy is embedded in the pathophysiology of the disease. According to Biel and Rao, mitophagy is induced solely in the aggressive breast cancer cell line MDA-MB-231 and not in the primary mammary epithelial cells (MCF-12A). Sub-lethal concentrations of TPP-conjugated redox molecules, including mitoquinone (MitoQ) and mitoApocynin (MitoA) exploit the increased Δψ_m_ reported in aggressive cancer cells leading to sustained mitophagy [3]. The authors elegantly provide evidence for the mitophagy-mediated resistance to chemotherapy that occurs via the canonical PINK1-Parkin pathway [3]. Interestingly, by playing with chemical inhibitors of the autophagy cascade, they detect the presence of basal mitophagic activity in MCF-12A, absent in MDA-MB-231.

This work re-invigorates the field of mitophagy in the progression and management of tumours. Discovering whether mitophagy induction is a feature of less aggressive cancer cells could unveil the culling of non-abiding mitochondria as a mechanism to reprogram cells towards metastatic tendencies. Conclusively, this research brings into focus mitochondrial life-cycle modulation as a key effector in cancer cell divergence and reveals therapeutic implications for the pharmacological fine-tuning of mitophagy to overcome chemotherapy resistance [7].
